# Subconscious value influences on science

**DOI:** 10.1016/j.shpsa.2025.102079

**Published:** 2025-10-21

**Authors:** Kevin C. Elliott, David B. Resnik, Wendy Lipworth

**Affiliations:** a Lyman Briggs College, Department of Fisheries and Wildlife, Department of Philosophy, Michigan State University, East Lansing, MI, 48825, USA; b National Institute of Environmental Health Sciences, National Institutes of Health, Research Triangle Park, NC, USA; c Department of Philosophy, Macquarie University, Australia

## Abstract

Philosophical scholarship on science and values has gradually shifted away from asking whether values have any legitimate role to play in scientific judgment and decision-making and toward considering how to responsibly manage value influences to protect the integrity, rigor, reliability, and trustworthiness of science. This scholarship has focused primarily on helping individual scientists deal with cases in which they are aware of the values at stake and are able to make conscious choices about whether to incorporate them into their judgment and decision-making. This means that accounts of the relationship between science and values tend to focus on *consciously perceived* values and value influences and tend to overlook the effects of *subconscious* values on scientific judgment and decision-making. In this paper, we aim to show how greater attention to subconscious value influence on judgment and decision-making can deepen our understanding of the relationship between science and values and provide useful guidance for managing value influences. To achieve our goal, we first examine the literature on values, interests, and conflicts of interest (COI) to demonstrate the potential for values to be subconscious and/or to exert subconscious influences on scientific judgment and decision-making. Next, we discuss some of the specific ways those subconscious influences could affect scientific reasoning. Finally, we show that most contemporary proposals for managing values in science are not well-suited to handling subconscious values or value influences, and we briefly point to some management strategies that merit further development.

## 1. Introduction

Philosophical scholarship on science and values has gradually shifted away from asking whether values have any legitimate role to play in scientific judgment and decision-making, and toward considering how to responsibly manage value influences to protect the integrity and trustworthiness of science (Holman & Wilholt, 2022; Resnik & Elliott, 2023).^1^ Major strategies that have been proposed for managing values in science include choosing the “right” values to influence science, limiting the “roles” of values in scientific judgment and decision-making, fostering openness about the influences of values, and ensuring that the scientific community is equipped to critically examine value influences (Elliott, 2022b).

A key feature (and we believe a limitation) of these strategies is that they tend to focus on managing circumstances in which scientists are consciously aware of the values they hold and the influences of these values on their decision-making rather than on *subconscious* values and value influences. Of course, subconscious values and value influences have not been completely neglected. Much of the feminist scholarship that helped to launch the contemporary literature on science and values highlighted the potential for androcentric values to influence science in both conscious and subconscious ways (see e.g., Hrdy, 1986; Longino, 1990; Solomon, 2001). Moreover, some figures, such as Eric Winsberg, have strongly emphasized that values are often present in the “nooks and crannies” of scientific practice, where they are not subjected to conscious evaluation (Winsberg, 2012; Winsberg, 2025; see also Ward, 2021).

Because of the methodological difficulties involved in distinguishing between conscious and subconscious value influences in science, it is difficult to arrive at precise conclusions about the extent of these influences across different aspects of science and fields of science. There is, however, empirical evidence from psychology and social science (which we describe below) suggesting that many values and value influences in science are likely to be subconscious. Despite this evidence, subconscious value influences have not been the focus of most recent philosophical literature, especially when it comes to the strategies that have been proposed for managing values (although see Winsberg, 2025).

One context in which the importance of subconscious values and value influences has been recognized is in the literature on interests and conflicts of interest in science. In that literature, there has long been discussion about the wide range of financial and non-financial concerns, preferences, and desires (i.e., interests) that scientists can have, and how these influence the scientific process. Importantly, those studying conflicts of interest (COIs) in science have observed how both interests and their influences are often subconscious (and, in some cases, frankly denied) (Moore et al., 2010; Resnik, 2023).^2^ As we will explain later, interests can be conceptualized as *contextualized values*, which means that subconscious interests offer a fruitful lens through which to explore the effects and management of subconscious values and value influences.

In this paper, we will draw upon insights from this literature on interests and COI to enrich the philosophical debate about science and values. In Sections 2 and 3, using subconscious interests as an exemplar, we will highlight the potential for values to exert subconscious influences on scientific judgment and decision-making. In Section 4, we will clarify in greater detail how these subconscious value influences might relate to scientists’ conscious reasoning processes. Finally, in Sections 5 and 6, we will show that most contemporary proposals for managing values in science are not well-suited to handling such subconscious influences, and we will briefly sketch an initial set of management strategies that could prove fruitful for addressing the subconscious influences of values in science.

## 2. Values and interests

### 2.1. Defining ‘values’

To begin this analysis, we need to explain what we mean by values and how they relate to interests. Two important distinctions from moral philosophy are relevant here. The first is the distinction between *descriptive* (factual) and *normative* (prescriptive) senses of value. In the descriptive sense, a value is something that is valued by people, that is, something *to which people assign positive significance* and therefore *prefer or desire* (Moore, 1903). For example, if we say, “most people value happiness,” we would be using value in the descriptive sense. But the fact most people value happiness leaves open the question of whether people *ought* to value happiness; that is, whether happiness is morally *good* or *worthy* of being valued. These questions relate to the normative, not descriptive, sense of value (Moore, 1903; M. Schroeder, 2021).

The second distinction is between *intrinsic* and *extrinsic* (or instrumental) value (Zimmerman, 2019). Something has intrinsic value if it is valuable (worthy of value) for its own sake, and not only for the sake of something else; something has extrinsic value if it is valuable for the sake of something else. Since antiquity, philosophers have proposed that many different things have intrinsic value, including happiness, knowledge, virtue, justice, health, pleasure, dignity, self-respect, love, non-human species, wilderness, and ecosystems (M. Schroeder, 2021). Some things, such as painful dental procedures, are regarded by most people as having only extrinsic value (that is, a means to dental health). Other things, such as health, might be regarded as both intrinsically valuable and a means to obtaining other values, such as happiness or pleasure.

Unfortunately, the notion of a ‘value’ is not as well-defined in the philosophy of science literature as it is in moral theory (see e.g., Biddle, 2013; Elliott, 2022b; Hilligardt, 2022; Ward, 2021). Elliott and Korf (2024) have recently proposed that there at least four concepts of “value” that have been employed in the philosophy of science literature.^3^ First, values can be understood as criteria or standards that guide scientific judgement and decision-making related to choosing theories or hypotheses, analyzing data, or interpreting data. Kuhn (1977), McMullin (1983), Kitcher (1993), and many other philosophers have argued that epistemic values, such as empirical support, simplicity, accuracy, fertility, coherence, and explanatory power, play a key role in scientific judgment and decision-making (Turri, 2021). Second, values are sometimes understood more broadly to encompass any causal factors that influence scientists’ reasoning. According to Miriam Solomon:

“‘Value’ has been used to include political values, aesthetic preferences, psychological biases, cognitive goals, personal and societal goals, ideologies, and pre-theoretic intuitions …. ‘Values’ include pre-theoretic assumptions, ethical conduct of inquiry, and causes of preference for one theory over another”

(2012, 332–333)

Third, values can be regarded as beliefs or attitudes about what is desirable. It is common for social scientists to define values in this way (e.g., Dietz, 2013), and it accords with the tendency of the contemporary values-in-science literature to focus on ethical values like public health or environmental sustainability (e.g., Brown, 2020; Douglas, 2009; Elliott, 2011b). Fourth, values can be regarded as desirable things themselves (i.e., things in the world) rather than people’s beliefs or attitudes about those things (see e.g., Elliott, 2017).

### 2.2. Defining ‘interests’

The word ‘interest’ is similarly ambiguous and has its own long history spanning philosophical, sociological, and economic scholarship. Interests have been understood as stakes (where an entity has an interest in something if it impacts their wellbeing), as sources of cognitive curiosity (“John is interested in medieval history”), and as motivators of behavior. With regard to the latter, there has been debate since the 16th Century about whether interests are the rational counterpart to the (destructive) “passions” or drivers of all human behavior, whether rational or irrational.^4^

These historical debates remain salient today, with the word ‘interest’, like the word ‘value’, being understood in both descriptive and normative senses (Gewirth, 2001). In the descriptive sense, an interest is something that *someone prefers or desires*. If we say that “John is interested in building his body,” we are saying something descriptive (or factual) about John. This is very similar to saying “John values body building.” However, there is also a normative sense of ‘interest’, which continues to play an important role in moral and political theory. An interest, in this sense, is something that is *beneficial (or good)* for a person (e.g., it is in this child’s interest to have this surgical procedure) (Braybrooke, 2001).^5^ Often, these two senses of interest coincide; for example, when a person wants to eat food, and the food is good for them. But they might not coincide, for example, when a person is not interested in quitting smoking, but quitting smoking is in their interests, or when John’s body building encourages him to abuse steroids. If interests are understood normatively, there are also situations in which something is in the interest of someone who is not conscious at all. For example, we might also say that it is in a fetus’s interests to not be exposed to dangerous toxins in utero. It also worth noting that we can apply the word ‘interest’ to groups of people, organizations, and even nations. For example, we could say that “General Motors has an interest in making a profit” or “foreign policy protects national interests.”

It is also important to note that interests can be *self-oriented* or *other-oriented*. For example, a scientist may have interests in earning money and advancing their career (self-oriented) and protecting the integrity of their profession and advancing human knowledge (other-oriented). It is often the case that self- and other-oriented interests coincide; for example, when a scientist makes an important discovery that advances their career (Hull, 1990). However, problems can arise in science (as we shall soon see) when self-oriented interests and other-oriented interests do not align. Problems can also arise when different other-oriented interests lead in conflicting directions.^6^ In both cases, one can be said to have a ‘conflict of interest’ (COI).

COIs are an ethical concern in scientific research because they can compromise scientists’ judgment and decision-making and reduce their willingness or capacity to fulfil their ethical, legal, or professional obligations or duties. This, in turn, increases the risk that they will commit research misconduct (such as data fabrication or falsification), engage in biased research practices (such as deceptive use of statistics or over-interpretation of data), or otherwise deviate from good research practices (Krimsky, 2003; Michaels, 2008; Oreskes & Conway, 2011; Shamoo & Resnik, 2022).

Importantly, the biases that result from COIs can be subtle. For example, in the conduct of clinical trials, COIs can lead researchers to design studies in ways that are superficially methodologically robust but increase the likelihood of a favorable result—e.g., by choosing particular outcome measures, comparators, or study duration (Krimsky, 2003; Lexchin, 2012). In this regard, dozens of studies have demonstrated statistically significant associations between sources of funding (and associated financial interests) and research outcomes in various domains of research, including medicine, pharmacology, nutrition, agriculture, public health, and environment (e.g., Barnes et al., 1995; Bekelman et al., 2003; Diels et al., 2011; Fabbri et al., 2018; Fanelli et al., 2017; Friedberg et al., 1999; Friedman & Friedman, 2016; Friedman & Richter, 2004; Mandrioli et al., 2016; Shrader-Frechette, 2011; Stelfox et al., 1998). This threatens not only the validity of research but also the perceived trustworthiness of research conducted by a person with a significant COI. This is not to say, of course, that having a COI can be equated with misconduct or bias, since a person with a COI could perform excellent research. However, a COI is a risk factor for misconduct, bias, and other ethical and methodological problems in research (Shamoo & Resnik, 2022).

In response to concerns about COI in science, most funding bodies, journals, and ethics committees have policies to guide their disclosure and management. In this policy context there is a tendency to ask for disclosure of the *kinds of circumstances* that give rise to interests rather than the interests themselves (as we define them above). For example, the International Committee of Medical Journal Editors (ICMJE) COI disclosure form, which is used by hundreds of biomedical research journals, lists various “interests” that should be disclosed, including grants, contracts, funding support, payments for lectures, consulting, expert testimony, attending meetings, ownership of stock, and patents and royalties (ICMJE, 2021). Likewise, the National Institutes of Health’s (NIH, 2024) policy on COIs lists items that constitute COIs, such as ownership of stock, patents, and payments for speaking or consulting,

In these policy contexts, there is no clear distinction drawn between the descriptive and normative sense of interest. This distinction is also absent in academic discussions. For example, in a highly cited paper on COIs, Dennis Thompson (1993, p. 573) defines a COI as “a set of conditions in which professional judgment concerning a primary interest (such as a patient’s welfare or the validity of research) tends to be unduly influenced by a secondary interest (such as financial gain).” Thompson (1993, p. 573) does not distinguish descriptive and normative senses of ‘interest’ and argues that a person could have primary interests in “the health of patients, the integrity of research, and the education of students.” Similarly, Shamoo and Resnik’s (2022) influential textbook, *Responsible Conduct of Research*, does not clearly distinguish between the descriptive and normative sense of interest.

Despite the ambiguity of these definitions and policies, analysis of how the term interest is *actually used* in this context reveals that it is *always used in the descriptive sense*—i.e., the interests that are of concern when it comes to COIs are things that are preferred or desired by a person, group of people, organization, or corporation, or are a means to something that is preferred or desired. It may also be the case that these interests are worth desiring, but it must at least be the case that the interest is actually preferred, desired, wanted, etc., because what matters in the context of COI management is what motivates people, not what is objectively good for them. Importantly, an interest may exist because an individual prefers or desires it, or because of the norms of a group of which they are a part, and an interest may exist at the collective level even if a particular individual does not value it. For example, the other-oriented interest of conducting scientific research with integrity cannot be said to be held by all individual scientists, as there are a small number who appear to care little for integrity. However, they still have an other-oriented “interest” in acting with integrity because they belong to a group for whom this is a strongly held (descriptive) norm according to which it is to be desired or preferred; and they still have a COI if they have a self-oriented interest that encourages them to act contrary to the norm of integrity.

### 2.3. Interests as contextualized values

If we view interests as things that are (descriptively) preferred or desired by individuals or groups, then interests clearly have a very close relationship with values. They most obviously coincide with Elliott and Korf’s (2024) third and fourth concepts of values: beliefs/attitudes about things that are good for us, or those good things themselves. Thus, interests are similar to social scientist Miroslaw Karwat’s characterization of values as “ideas of needs,” or expressions of a “subject’s attitude toward its own needs” (1982, 198). One could easily replace “needs” with “interests” in Karwat’s definition.^7^

**Fig. 1.**
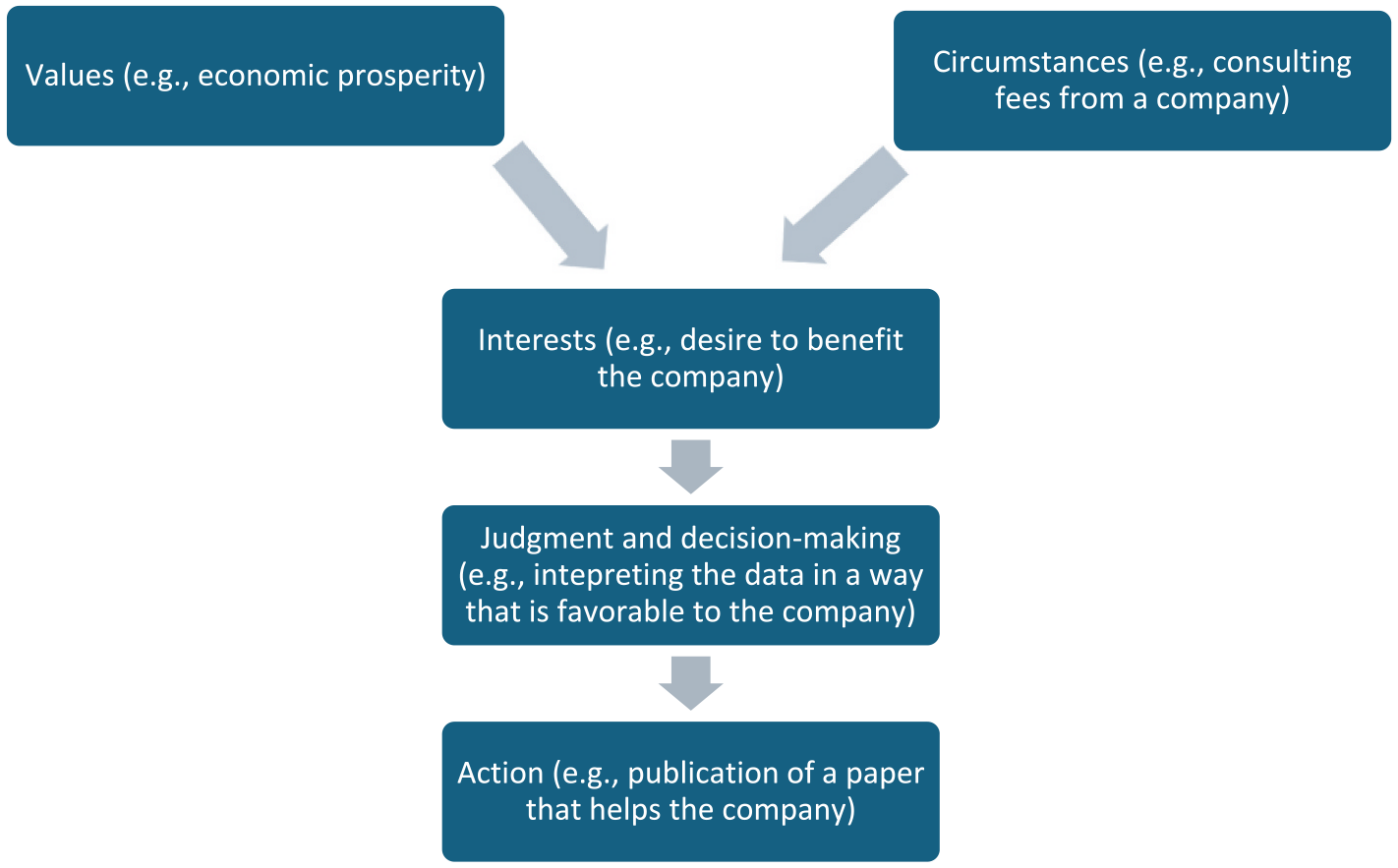
Values, interests, judgment/decision-making, and action.

While we think that values and interests are closely related, we think that values are often more abstract than interests. More specifically, *we propose that an interest can be understood as a contextualized (or concretized) value.* That is, an interest is a value that exerts an influence or operates within a particular social, institutional, political, or economic context. (See Table 1 for examples of values, interests, and the circumstances that generate the latter.) For example, when someone receives consulting or speaking fees from drug companies or owns patents on therapeutic compounds, the abstract value of economic prosperity becomes contextualized and concretized as an interest in maintaining a relationship with a company (so that one continues to be called upon as a speaker) or seeing a company succeed financially (so that one can reap the associated financial rewards). This may, in turn, encourage the person to promote the success of the company, and their own wellbeing, by biasing data that pertains to the company’s products (Resnik, 2007; see Fig. 1). Similarly, someone who is doing a health economic analysis of a drug that could be used by a family member may be more inclined to find data supporting its cost-effectiveness if they have a family member who could benefit from insurance coverage. This interest is a contextual instantiation of the more abstract value of health.

This distinction between (abstract) values and (contextual) interests can help to explain, in part, why those who are concerned about COIs require scientists to disclose interests but not values. First, because interests are contextual, they distinguish people from one another. For example, while most people value economic prosperity, far fewer will be consultants to pharmaceutical companies. Additionally, because interests are more concrete, they can give us more information about how people are likely to think and behave. Simply saying that someone values economic prosperity does not tell us much about where their concerns and loyalties are likely to lie, whereas it seems reasonable to assume that someone with shares in a company will tend to act in a manner that benefits the company.

This discussion of interests can enhance the understanding of values in the recent literature on values and science in at least two ways. First, it shows how this literature could expand to focus on a wider array of values than it typically emphasizes. Most recent work on science and values has focused on two sets of values listed in Table 1: economic prosperity and moral, political, or social concerns (see e.g., Brown, 2020; Douglas, 2009; Douglas, 2021; Elliott, 2017; Steel & Whyte, 2012). The literature on COIs highlights other values that also merit consideration, such as career-oriented values and those associated with professional and personal relationships (see Table 1). Second, this discussion shows how the literature on values and science could consider the influences of values more concretely by focusing more attention on interests. For example, even though it may be helpful in some cases to consider the ways in which the general value of economic growth could influence scientists, it may be more helpful in other cases to consider how that value is contextualized for specific scientists in the form of interests that they have by virtue of their employment or funding from specific companies. Similarly, in addition to considering the ways in which the general value of environmental sustainability could influence scientists, it is also important to consider how that value is contextualized for specific scientists in the form of interests that they have by virtue of their leadership or consulting for environmental organizations.

As philosophers of science move forward to consider how values can become contextualized in the form of interests, we think it is important to comment on the distinction between financial and non-financial interests and COIs. The COI literature distinguishes between financial interests (such as stock or speaking fees) and non-financial interests (such as personal relationships or political activity) (Resnik, 2023; Wiersma et al., 2024). Some COI policies require authors to disclose *all* interests that could bias their research, while others only require authors to disclose *financial* interests that could bias their research (Resnik et al., 2017). Some commentators argue that COI policies should not treat non-financial interests as contributing to COIs, because: 1) non-financial interests have less of an impact on science than financial ones; 2) non-financial interests are difficult to measure and quantify; and 3) everyone has some type of non-financial interest (Bero & Grundy, 2016). Although we will not take a stand in this paper concerning non-financial COI policies (see Resnik, 2023; Wiersma et al., 2018), we would like to note that in our analysis it makes little difference whether an interest is financial or non-financial, since in either case it is an instantiation of some type of value (i.e., economic, personal, professional, etc.).

## 3. Subconscious values and interests

Recognizing that both values and the subset of values we refer to as interests can be understood as beliefs or attitudes about things that are preferred or desired by individuals or the groups to which they belong, the next step is to consider how they affect us. As noted earlier, psychologists, social scientists, and critics of science (and scientists themselves) have long observed how both interests and their influences are often subconscious. In what follows we provide several lines of evidence to support this claim. In doing so, we do not aim to provide a definitive argument establishing that all or even most interests and their impacts are subconscious; rather, we merely aim to show that given our current psychological understanding, it is plausible that at least some interests, and therefore at least some values, exert significant subconscious effects on people’s reasoning (Resnik, 2007, 2023; Kafaee et al., 2021, 2022).

Evidence for the subconscious nature of interests and their influences can be drawn from general psychological theories as well as psychological research specifically on the topic of interests and COIs. From a general psychological perspective, a particularly significant phenomenon of relevance to interests and COIs is motivated cognition (see e.g., Dunning, 1999). This refers to influences of people’s motivations (e.g., their goals or desires) on their judgment, decision-making, or reasoning. Significantly, motivated cognition occurs not merely through conscious cognitive processes but also through subconscious processes. For example, cognitive dissonance theory provides an example of motivated cognition that can often play out subconsciously (Harmon-Jones & Mills, 2019). According to cognitive dissonance theory, when people have two cognitions (e.g., a value and a belief) that conflict with each other, they will tend to take on new cognitions that are more congruent, such as by rejecting or revising one of the original cognitions. Another relevant example of motivated cognition is confirmation bias, i.e., the tendency to look for evidence to support something that one expects to believe or already believes while ignoring or downplaying evidence against it (Tversky & Kahneman, 1974; Klayman, 1995; Sutton, 2024). Confirmation bias, which also operates at a subconscious level and affects many aspects of human cognition and perception, has direct implications for the link between interests and epistemic behavior, since interests create expectations or inclinations to believe things (Kafaee et al., 2021, 2022). Both cognitive dissonance dynamics and confirmation bias might manifest themselves, for example, when a scientist is inclined to believe a hypothesis and then tends to ignore evidence against it because the truth of the hypothesis would help them obtain funding, recognition, or career advancement (Santos, 2021; Shamoo & Resnik, 2022).

In addition to this general evidence from psychological theory on motivated cognition, there is some psychological research more specific to COIs that shows that interests, conflicts of interests, and their influences are often subconscious. For example, Don Moore and his colleagues have performed several experiments to study the psychological phenomena associated with COIs (see e.g., Chugh et al., 2005; Moore & Loewenstein, 2004; Moore et al., 2010). An important theme of their research is that once people have been placed in “partisan roles” (which included both financial and non-financial relationships in their experiments), it is difficult for them to “extricate themselves from the influence” of those roles, even if they have the goal of being impartial (Moore et al., 2010, p. 46). Notably, when Moore and his collaborators offer advice for policy makers who seek to address the influences of COIs, they draw on their empirical findings to emphasize that it is a mistake to think that “people are aware of the degree to which selective mental accessibility of thoughts, evidence, and arguments can influence their professional judgments” (Moore et al., 2010, p. 46). Instead, they emphasize that policy responses must take account of the psychological research indicating that “biased information processing is not only pervasive, but is typically unconscious and unintentional – i.e., seldom a matter of deliberate intentional choice” (Moore et al., 2010, p. 46). Paul Thagard (2007) argues for the same conclusion—i.e., that the effects of COIs are largely subconscious—by drawing on psychological research indicating that human decision-making relies on an integration of both cognitive and emotional processing that is not entirely conscious.

Another body of psychological research with relevance to COI in science concerns the “gift relationship” (Titmuss, 2018). This describes a psychosocial phenomenon in which we are motivated by altruism to act in the service of others, and also to reciprocate for gifts that we receive. When a person receives a gift, they feel a desire or sense of obligation to reciprocate even if the giver did not expect them to do so, because doing so signals appreciation, strengthens social connections, and/or enables them to avoid feeling indebted or appearing as if they are “free riding.” This provides an explanation for the finding demonstrated in numerous studies that gifts, payments, and other interactions with pharmaceutical companies can influence physicians’ prescribing behavior, even when gifts are small and physicians claim that they are not aware of acting under this influence or flatly deny that money affects them (Chren & Landefeld, 1994; Katz et al., 2003; Mitchell et al., 2021; Orlowski & Wateska, 1992; Perlis & Perlis, 2016; Wood et al., 2017). This finding is potentially relevant to science both because receipt of funding or payment for consulting might be experienced as gifts and because scientific hypothesis testing involves similar cognitive activities to clinical prescribing, such as information processing, perception, and judgment. In both contexts, a subconscious “gift relationship” is set up through which interests can affect judgment and decision-making at a subconscious level.

Partly because of the recognition that COIs can exert significant subconscious effects of which scientists are not aware, COIs are generally defined in scientific contexts as a *set of circumstances* that *creates a risk* of biased judgment or decision-making and not *an action involving intentional bias* (Institute of Medicine, 2009). It is simply too difficult to expect scientists to recognize how their interests might have influenced them. Moreover, it is not only the *influences* of interests and COIs that can be subconscious, but in some cases also the *interests themselves*.

It is likely for this reason that those who request disclosure of interests often ask for disclosure not of the interest itself, but of the circumstances in which the interest arises (which is often then referred to in policy documents as the ‘interest’); although this is conceptually problematic, it is probably wise given how difficult it can be for people to recognize or even acknowledge that they have relevant context-specific interests (see Table 1). In this regard, the distinction between financial interests/COIs as opposed to non-financial interests/COIs also becomes relevant because people may be even less likely to recognize their non-financial interests than their financial interests.

The distinction between interests and their influences should be kept in mind when considering strategies for managing interests and values in science, which we will turn to later. Some management strategies may be designed primarily to make scientists more aware of their subconscious interests (and the circumstances that have given rise to them), whereas other strategies may focus on addressing the subconscious *influences* of those interests, whether or not the interests are brought to conscious awareness. The literature on COI focuses primarily on the subconscious *influences* of interests, but the dangers of COIs are exacerbated when people do not even consciously recognize the interests themselves.

Importantly for our argument, the points that we have made here about the subconscious influences of interests arguably apply to values more broadly. We have used the literature on interests and COIs to motivate our discussion because their subconscious influences have been studied relatively closely and extensively. However, we have argued that interests are a kind of value, so the phenomena that we have described for interests also obviously apply to at least some values. We have already noted that the terminology of ‘values’ can be applied to a wide variety of phenomena in the philosophy of science, so different kinds of values could vary in the extent to which they display subconscious influences. However, to the extent that not only interests but also values in general (including the ethical or political values commonly discussed in the values-and-science literature) involve what is *desired or preferred*, they are likely to trigger motivated cognition, which consists in “the pervasive tendency to think in ways that produce conclusions consistent with one’s desires” (Lemay & Clark, 2015).

## 4. Understanding subconscious value influences

Having established that values plausibly influence scientists’ judgment not only consciously but also subconsciously, let us consider in more detail the specific avenues through which these influences may occur and how they might relate to the conscious value influences that have been previously emphasized by philosophers of science. This section begins with a theoretical account of how subconscious value influences could occur and then considers how these influences could play out in a concrete case study. Our fundamental claim in both the theoretical account and the case study is that values are likely to influence scientists subconsciously whenever scientists face contingent choices, like which questions to ask, what study designs to implement, what concepts or categories to employ, how to analyze data, how to interpret results, what standards of evidence to require in order to draw conclusions, and how to frame findings (e.g., Brown, 2020; Elliott, 2017). Philosophers of science have previously emphasized that scientists can *consciously* bring epistemic and non-epistemic values to bear on these choices (Biddle, 2013), but values likely also *subconsciously* influence how scientists weigh and interpret both evidence and conscious values in these decision contexts. In other words, one could think of subconscious value influences shaping the cognitive environment in which scientists make conscious judgments concerning their work. Thus, even when scientists believe they are making conscious value judgments, subconscious values could still be influencing their epistemic conduct, especially when there are significant outcomes at stake.

Consider, for example, a case in which a scientist thinks that one interpretation of their results fits better with some of their data, whereas another interpretation fits better with other data. Judgments about which interpretation to adopt can be very difficult to make, and different scientists frequently disagree about such judgments. Therefore, it is highly plausible that values could subconsciously influence which judgment a scientist in this situation finds to be most plausible. For example, if the scientist held strong values in favor of environmental sustainability, and one interpretation of their data appeared to be more protective of the environment, they might *subconsciously* perceive that interpretation to be more compelling. The scientist might try to limit the potential for subconscious influences by *consciously* appealing to specific epistemic or cognitive values to determine the best interpretation. For example, they might examine whether one set of data was more relevant, more reliable, or more compatible with additional lines of evidence—and therefore they could conclude that the interpretation that fits better with that data set should be preferred. Nevertheless, this merely creates a regress because the judgments about which data set is most relevant, reliable, or compatible with other lines of evidence is still likely to be *subconsciously* influenced by the scientist’s environmental values.

This example is likely generalizable to any circumstance in which the literature on science and values has called for conscious judgments involving values because, in all cases, there are opportunities for subconscious value influences on those judgments. For example, when scientists face situations in which it is unclear which choice is best from a purely epistemic perspective, many figures in the science-and-values literature advise them to consciously make a choice that accords with important non-epistemic values (see e.g., Biddle, 2013; Steel & Whyte, 2012). However, values are highly likely to subconsciously influence whether scientists assess two choices as being epistemically equivalent. For example, one scientist might think that two interpretations of the available data are epistemically equal (and therefore it would be appropriate to appeal to non-epistemic values to settle the matter), while a different scientist subject to different subconscious value influences might think that one interpretation of the data is clearly epistemically superior. Similarly, an important theme of the science-and-values literature is that scientists should manage inductive risk throughout their work by making choices that appropriately balance the costs of false-positive and false-negative errors (Douglas, 2009, 2021). Once again, scientists are likely to be subject to subconscious value influences when deciding what methodological choices generate an appropriate balance between those costs (e.g., is it most appropriate to demand a 95% statistical significance level for studies in a particular research context, or should it be 90%, or 99%?).

With this theoretical account in mind, we can consider a concrete case study. Over the past two decades, different groups of scientists have disagreed strongly about the topic of endocrine disrupting chemicals (EDCs). EDCs interfere with the hormonal system, thereby potentially causing cancers and reproductive, developmental, and/or neurological problems at particularly low doses (Colborn et al., 1993; Kahn et al., 2020). However, the extent to which particular EDCs actually generate harmful effects on humans at low dose levels has been sharply debated (Elliott, 2011b; McIlroy-Young et al., 2021; Myers et al., 2009; Tyl, 2009). For example, many academic scientists have argued for years that bisphenol A (a chemical used in many products, including can liners and receipts) was likely to be harmful to humans at current levels of exposure, whereas scientists working with the U.S. Food and Drug Administration (FDA) and the European Food Safety Authority (EFSA) have rejected this conclusion (Resnik & Elliott, 2015; Vandenberg & Prins, 2016).^8^

A striking feature of these debates is that scientists affiliated with the chemical industry tend to be particularly skeptical about the view that EDCs could be causing significant harm at low doses, while scientists affiliated with environmental organizations tend to accept this view, and academic scientists fall somewhere between these extremes (Elliott, 2011b). This is surely at least partly a function of the different epistemic and non-epistemic values held by the scientists and other leaders associated with these organizations. These differing values could be generated by many different factors, including self-selection by scientists who are more comfortable working with industry or environmental organizations, selection by industry or environmental organizations of the kinds of scientists they want to employ, bandwagon effects caused by spending time with others with similar views, and interests associated with the financial success and social reputation of the organizations with which scientists work.

There are a wide variety of contingent choices involved in assessing the evidence for low-dose harmful effects from EDCs. For example, when scientists see data that appear to indicate harmful effects of EDCs at low doses, they must assess whether the data are reliable or whether they could be caused by weaknesses in experimental design. In addition, they must decide whether harmful effects on specific endpoints (e.g., organ weight) are likely to translate into harmful effects for the whole organism. They also sometimes must weigh conflicting evidence from different studies, with some studies appearing to provide evidence for harmful effects and other studies appearing to provide evidence of safety.

Given the theoretical account provided previously in this section, it is easy to see how differing values could influence the contingent choices made by scientists working on EDCs not only in a conscious manner but also subconsciously. For example, an industry scientist might be subconsciously inclined to regard low-dose data points that indicate EDC toxicity as unreliable, whereas a scientist working for an environmental organization might be subconsciously inclined to regard those low-dose data points as reliable. Of course, they are likely to make conscious appeals to various forms of evidence and epistemic values in support of their positions about the reliability of the data. In fact, the scientists involved in these debates typically insist that there are strong epistemic reasons in support of their positions (Resnik & Elliott, 2015; Vandenberg & Prins, 2016). However, it is highly likely that subconscious non-epistemic value influences are among the factors that “tip the scales” and influence how these scientists weigh and assess the available evidence and epistemic values.

Before concluding this section, it is important to consider a potential objection. One might think that even if these subconscious value influences occur, they are unproblematic (and therefore do not need to be managed) as long as they do not cause scientists to violate the norms of their scholarly communities.^9^ We disagree with this objection. The burgeoning literature on managing values in science is not concerned solely with avoiding cases in which values generate “bad” science that violates scientific norms. After all, a crucial worry about the value-ladenness of scientific research is that it could threaten the autonomy of decision makers if they do not recognize that the values underlying a body of scientific work fail to match their own values (Betz, 2013; John, 2025; Lusk, 2021). This threat to the autonomy of decision makers is a problem whether or not the science at issue violates fundamental norms of science. For example, even if none of the scientists involved in research on EDCs is violating fundamental community norms, it is still very challenging to make thoughtful policy decisions when the scientists working on EDCs hold such wildly divergent views. Gürol Irzik and Faik Kurtulmus’s distinction between “basic” epistemic trust (where scientists can be trusted to follow basic standards of good science) and “enhanced” epistemic trust (where scientists can be trusted to make methodological decisions in ways that accord with the values of those using the science) helps to clarify the problem (Irzik & Kurtulmus, 2019). Decision makers might be able to have basic trust in a scientist’s work as long as they know that the scientist is not violating fundamental community norms, but they probably cannot have enhanced trust if they fear that a scientist’s sub-conscious values could be steering the research in ways that differ from the values of the decision maker. Therefore, it is important to explore ways to identify, communicate about, and potentially limit subconscious value influences on topics like EDCs whether or not those influences violate community norms. This is the task to which we turn in the next section, where we contend that most of the strategies that philosophers of science have proposed for managing the influences of values do not succeed at addressing the *subconscious* influences of values.^10^

## 5. Managing subconscious value influences

Holman and Wilholt (2022) have helpfully organized value management strategies into a taxonomy consisting of five major categories: axiological, functionalist, consequentialist, coordinative, and systemic. Elliott and Korf (2024) have argued that it is important to consider how different categories of management strategies may be more effective for managing different kinds of values (i.e., criteria, causal influences, beliefs/attitudes, or states of affairs). Here, we consider how different categories of management strategies may be more or less effective for managing *subconscious* values and value influences. We conclude that only the systemic approach is well-designed for managing subconscious values and value influences, and even it needs to be strengthened and developed further.

### 5.1. Axiological management strategies

Axiological strategies focus on ensuring that only an appropriate set of values influences scientific work. These appropriate values might be identified through ethical reasoning (e.g., Brown, 2020; Kourany, 2010) or through a political process (e.g., Kitcher, 2001). Notably, the ethical and political processes associated with axiological management approaches tend to focus on conscious deliberation about value influences. For example, in Matthew Brown’s book *Science and the Moral Imagination* (Brown, 2020), his vision for managing values focuses on helping the members of scientific labs to engage in conscious and creative ethical reflection about how they can do their work in a way that respects relevant values and stakeholders (Brown, 2020, p. 228). Admittedly, he recognizes that scientists frequently make choices without recognizing how values can be implicated in those decisions, and thus he recommends creating interdisciplinary collaborations with humanists or social scientists who can help bring their value-laden decisions to light. Thus, he provides some insights relevant to addressing subconscious value influences (to which we will return in the next section), but his primary strategy for managing them relies on consciously deliberating about them.

An emphasis on conscious value influences is also evident in contemporary efforts to foster a “political” or “democratic” approach to managing values in science. In his classic book, *Science, Truth, and Democracy*, Kitcher (2001) called for value-laden decisions in science (specifically, decisions about what projects to undertake) to be based on the conclusions that well-informed decision-makers would arrive at if they were placed in an ideal deliberative setting. More recently, Schroeder (2021) has been calling for scientists to handle value-laden choices in ways that align with the values of the public or its representatives, and Parker and Lusk (2019) have called for scientists to guide their work based on the values of end users (see also Thoma, 2024). All these proposals to make science responsive to democratic values appear to presuppose that scientists are able to consciously choose to guide their work in accordance with particular values.

These axiological strategies face obvious difficulties when managing subconscious values and value influences, both because scientists might not be aware of *which* values they hold and because they might not be aware of *how* those values influence them. Proponents of axiological strategies could respond that one component of their strategies is the effort to bring to consciousness values and value influences that have previously been subconscious. However, such efforts may not be successful if they rely on scientists’ own self-reflection and subsequent communication, as motivated cognition cannot easily be overcome on one’s own. Furthermore, even if scientists are able to make visible and communicate values that are then deemed to be “appropriate” through a legitimate ethical or political process, the axiological strategy could still fail as an adequate management strategy if the *influences* that the values exert remain subconscious and therefore fail to be appropriately recognized and communicated.

### 5.2. Functionalist management strategies

The second category of management strategies proposed by Holman and Wilholt (2022) are functionalist ones. These approaches limit the influences of values such that they are allowed to play only some roles but not others. The most influential functionalist approach is the one developed by Heather Douglas. In her important book, *Science, Policy, and the Value-Free Ideal*, Douglas (2009) rejects what she calls the value-free ideal (VFI), that is, the idea that the “value judgments internal to science, involving the acceptance and evaluation of scientific results at the heart of the research process, are to be as free as humanly possible from all social and ethical values” (Douglas, 2009, p. 45). A key argument for her position is that the decision to accept or reject a scientific hypothesis or theory involves inductive risk—that is, the risk that the decision could turn out to be mistaken. Scientists cannot ethically ignore this risk (Douglas, 2009), and they cannot delegate all decisions about inductive risk-taking to others without generating interpretive difficulties for them (see e.g., Elliott, 2011b; Havstad & Brown, 2017). Therefore, they must decide how much evidence is needed to accept or reject hypotheses, and this decision must take into account social and ethical values related to the consequences of being mistaken (Douglas, 2009, pp. 66–86).

Douglas (2009, p. 15) recognizes that allowing these values to affect scientific judgment and decision-making creates the risk that science will become corrupted. To prevent this from happening, she distinguishes between *direct* and *indirect* roles for values in science and argues that to protect the integrity and trustworthiness of science, values should play only an indirect role.^11^ According to Douglas, values play a direct role when they function as evidence and “act as reasons in themselves to accept a claim” (2009, 96). In contrast, values play an indirect role when they help “to decide what should count as *sufficient* evidence” for a claim (2009, 96, italics in original). For example, to protect public health, scientists could set the p-value at 0.03 for rejecting a null hypothesis that a drug is unsafe at a particular dose.^12^ In this case, a value—i.e., public health—would be playing an indirect role. However, if they rejected the null hypothesis in a particular study because they thought this would promote public health, the value would be playing a direct role.

Other functionalist approaches take slightly different approaches to delineating appropriate and inappropriate roles that values can play in science. For example, Steel and Whyte (2012) have argued that non-epistemic values can be problematic even when they play an indirect role. Thus, they have argued that non-epistemic values should influence scientific reasoning only when a choice is left underdetermined by epistemic values (Steel, 2017; Steel & Whyte, 2012). In this way, it is not possible for non-epistemic values to override epistemic values. Although Steel and Whyte (2012) claim that their approach is focused primarily on *types* of values (i.e., epistemic vs non-epistemic values) rather than *roles* for values, one could interpret their focus on not allowing non-epistemic values to override epistemic values as a functionalist approach. Holman and Wilholt argue that Elizabeth Anderson’s call for preventing values from driving inquiry to a “predetermined conclusion” (Anderson, 2004) is another functionalist approach. Finally, one could argue that proponents of the “value-free ideal” (VFI) also subscribe to a functionalist approach, insofar as they allow non-epistemic values to influence decisions about what questions to ask and how to apply scientific results for public policy making while excluding non-epistemic values from influencing core aspects of scientific reasoning (see e.g., Betz, 2013; Lacey, 2017).

As in the case of the axiological strategies, these functionalist approaches are not well suited for managing subconscious value influences because one cannot reliably determine what role, if any, subconscious values might be playing in scientists’ reasoning. For example, Douglas’s distinction between direct and indirect roles for values holds up only if scientists *consciously choose* to treat values as “reasons in themselves” (a direct role) or as reasons for setting standards of evidence in a particular way (an indirect role). If values influence scientists subconsciously, one cannot reliably determine whether values are playing one role as opposed to the other (see Bluhm, 2017; Elliott, 2011a; Steel & Whyte, 2012).^13^ Similarly, Steel and Whyte’s approach to managing value influences also relies on conscious choices made by scientists, since one cannot prevent non-epistemic values from overriding epistemic ones unless the value influences are conscious. The same difficulties hold for Anderson and for proponents of the VFI. If values influence scientists subconsciously, then they cannot reliably exclude the values from driving inquiry to a preferred conclusion or from influencing core aspects of scientific reasoning. For example, one can imagine a scenario in which a scientist tried not to allow their values to predetermine their conclusions, but their values nevertheless subconsciously exacerbated their existing confirmation biases so that the available evidence seemed to strongly support a particular hypothesis. The scientist might say they were not allowing values to predetermine their conclusions (because it seemed that way at a conscious level) even though the values actually did play that role at a subconscious level.

As with axiological strategies, proponents of functionalist strategies might argue that they assume that values and value influences that have previously been subconscious will be brought to consciousness prior to determining what role they will play. However, as noted above, this assumption that scientists can be made aware of subconscious values and their influences is highly dubious. At the very least, significant attention must be paid to the process of bringing these values and their influences to conscious awareness.

### 5.3. Consequentialist and coordinative management strategies

The third and fourth kinds of management strategies discussed by Holman and Wilholt (2022) are consequentialist and coordinative ones. These can be fruitfully considered together because the challenges associated with them when values are subconscious are very similar. Consequentialist strategies strive to achieve particular consequences or outcomes (e.g., aims that are chosen through a democratic process; Intemann, 2015) as a result of incorporating values in science. Coordinative strategies strive to align the influences of values with the expectations of those who will be using or receiving the research. So, for example, if a local community wanted to obtain predictions about climate change that erred on the side of predicting worst-case scenarios, the coordinative strategy would call for climate modelers to perform their work in ways that align with that value (see e.g., Parker & Lusk, 2019). Elliott and McKaughan (2014) have proposed a value-management approach that has characteristics of both the consequentialist and coordinative approaches. They argue that scientists need to consider the local aims associated with the research that they undertake in order to arrive at appropriate criteria for assessing their work. So, for example, if they are performing risk assessments to be used by regulatory agencies, then they need to consider not only the accuracy of their results but also the speed with which they can generate results and the extent to which their methods can be standardized. Therefore, they argue that transparency about values (i.e., the criteria used for assessing scientific work) is crucial in order to maintain the legitimacy of scientific research (see also Elliott & Resnik, 2014; Lusk & Elliott, 2024).

Unfortunately, consequentialist and coordinative management strategies are relatively unhelpful for addressing the subconscious influences of values. As is the case with axiological and functionalist strategies, in cases where scientists are not even aware that they hold particular values, they cannot reliably ensure that those values are contributing to preferred outcomes or that they are aligned with the expectations of those using their research. And even in cases where scientists know that they hold particular values, they cannot assess the impacts of those values on the outcomes of their research or its alignment with stakeholders’ values if the values exert subconscious influences. And, as with axiological and functionalist strategies, it cannot simply be assumed that subconscious values will be brought to consciousness and effectively integrated into reasoning and decision-making. Moreover, Elliott and McKaughan’s (2014) call for researchers to be transparent about the influences of values on their work runs into significant difficulties when they are not conscious of those influences.^14^

### 5.4. Systemic management strategies

This brings us to Holman and Wilholt’s (2022) fifth, “systemic” set of strategies for managing values. These approaches strive to *organize the scientific community* in such a way that any problematic influences of values at the level of the individual scientist are identified and critically assessed at the level of the community, even if the individuals who hold these values are not aware of the values or their influences. Helen Longino’s seminal work, Science as Social Knowledge (1990), was central both to launching the study of science and values and to promoting systemic approaches for managing values. In her book, Longino argues against the “myth of scientific value neutrality” (1990, 224), that is, the view that “science is free from personal, social, or cultural values” (1990, 4). Her main argument against this view is that scientific evidence, by itself, does not provide sufficient warrant for justifying hypotheses (or theories) because “the relation between hypotheses and evidence is determined by background assumptions operative in the context in which data are being assessed” (1990, 58). Longino argues that these background assumptions are unavoidably value-laden, in the sense that non-epistemic values (or what she calls “contextual values”; 1990, 4) both causally influence them and are affected by them. Therefore, she argues that the objectivity of science cannot be grounded in excluding values from the reasoning of *individual scientists*; rather, it must be a function of the *scientific community’s* structure and practices (1990, 74). Specifically, she argues that the scientific community should have four characteristics: recognized venues for criticism, shared standards of evaluation, uptake of criticism, and tempered equality of intellectual authority (Longino, 1990, 2002).^15^ Together, these characteristics foster objectivity by generating transformative criticism of background assumptions. Others, such as Miriam Solomon (2001), have developed similar proposals for structuring the scientific community so as to critically assess the influences of values.

This kind of approach holds greater potential than the others do for managing the subconscious influences of values. Even if one individual or group of scientists is not aware that they hold particular values or that their values are influencing them in particular ways, another group of scientists with different values has the potential to identify weaknesses in the first group’s reasoning (which could be the result of various subconscious value influences). Importantly, this identification of weaknesses in scientific reasoning could serve as a helpful management strategy even if specific subconscious value influences are never identified. For example, in the EDC case, scientists associated with environmental NGOs could potentially point out that industry scientists are dismissing a surprisingly wide array of low-dose data about harmful effects without providing much reason to think that the data are unreliable. The scientists from the NGOs might not need to speculate about the subconscious value influences underlying this reasoning in order to help manage those value influences. It might be enough just to help other scientists and policy makers recognize the weaknesses in the industry scientists’ reasoning so they can approach the industry scientists’ conclusions with appropriate caution. Thus, unlike the other management approaches considered in this section, the systemic approach can function even when values influence scientists subconsciously.

However, this does not mean that systemic management approaches are entirely effective in their current forms. If they are to serve effectively for managing subconscious value influences, care needs to be taken to ensure that the scientific community is indeed structured in a way that maximizes the potential to do this. So, for example, there needs to be an appropriate diversity of perspectives within the scientific community, and (as Longino noted) there need to be effective opportunities for critically evaluating scientific work. Moreover, it is still possible for subconscious value influences held by influential scientists to have a harmful impact on society. For example, Oreskes and Conway (2011) have argued that the strong free-market capitalist values held by prominent scientists like Fred Singer and Fred Seitz probably contributed (either consciously or subconsciously) to their skepticism of anthropogenic climate change. Unfortunately, even though the broader scientific community corrected the misleading claims made by these climate scientists, their value-influenced work was still very harmful. The fossil-fuel industry was able to trumpet the skeptical claims of these individuals as justification for blocking important policies to address climate change, and their skepticism also caused significant confusion for the public at large.

## 6. Managing subconscious value influences

The preceding analysis raises significant challenges for the current approaches to managing values in science. We have argued that values can have subconscious influences on scientists’ reasoning, and we have shown that most of the existing proposals for managing values are not designed to handle these influences. This does not mean that these proposals need to be abandoned; they may still be helpful for managing conscious value influences. Nevertheless, greater care needs to be taken to make visible and manage subconscious values and/or value influences.

We suggest that systemic approaches provide a good starting point for performing this task because they place the responsibility for value management on the scientific community rather than on individual scientists. Nevertheless, there are still numerous ways in which they can be strengthened and augmented with a specific focus on managing subconscious values and their influences. In this section, we briefly suggest five ways in which these approaches can be strengthened: (1) organized reflection; (2) open science; (3) effective peer review; (4) recusal and limitations; and (5) ethical, legal, and regulatory oversight. Interestingly, these suggestions accord broadly with the strategies that are commonly proposed to manage COIs. For example, on the basis of his analysis of the subconscious mechanisms underlying COIs, Thagard (2007) recommended that “when complete avoidance of conflicts of interest is not possible, the strategies of optimal reasoning patterns, disclosure, social oversight, and understanding of neuropsychological processes [to stimulate reflection on interests] may combine to reduce the prevalence of immoral decisions deriving from conflicts of interest” (Thagard, 2007, p. 378). This suggests that those managing COIs have an implicit, if not explicit, understanding of the subconscious nature of many values and their influences and that we can fruitfully integrate their insights into strategies for managing values in science.

The strategies described below are designed to help achieve a “two-tiered” approach to managing subconscious value influences. First, many of these strategies can help to bring subconscious values to the conscious awareness of either the scientists who hold them and are influenced by them, or to the awareness of other scientists who scrutinize their work. Second, given that these efforts to become aware of values will often fail and that the management of these values requires more than merely becoming aware of them, the strategies below can also help to constrain or guide their *influences*. This second tier of management is important because we have seen that people can be aware of their values while still being subject to subconscious influences from them, so it would be a mistake to depend solely on conscious value-management strategies. Admittedly, we are providing only a brief sketch of ways to strengthen systemic management approaches in this section; our goal is to spur further efforts to develop these approaches further.

### 6.1. Organized reflection

One important step for enhancing the effectiveness of systemic value-management approaches is to foster strategic forms of organized reflection on the values that could be impinging subconsciously on scientific work. In keeping with our two-tiered approach mentioned above, this kind of reflection can serve at least two purposes. In some cases, organized reflection could help scientists to recognize their values and disclose them to others so that they could be subjected to conscious value management strategies like the ones discussed in Section 5 (Tier one). Second, by recognizing the kinds of values that *could be at play* but that might not be made entirely visible, reflection could help the scientific community take more strategic steps to ensure that there is an adequate diversity of perspectives in place to address potential subconscious *influences* of those values (Tier two; see e.g., Solomon, 2001).

Organized reflection can be facilitated by routine disclosures of values, such as the disclosures of interests (or the associated conflicts of interest or circumstances that generate them) that are expected when research is funded, published or presented. As noted above, many journals use the ICMJE’s form for disclosing financial interests in scientific papers and in peer review. Resnik (2023) provides a list of important non-financial interests that authors, editors, and reviewers should disclose, including direct research interests. In addition to routine disclosures, more dynamic processes can also be put in place to support organized reflection, including within research teams and as part of the publication process. For example, ethnographic researchers are increasingly striving to foster reflection about the ways their own positionality, behavior, and interactions with research participants could affect their results (Büter, 2024). More broadly, scientists across a number of fields are encouraging the pursuit of reflexivity and the development of positionality statements in an effort to bring values to light (Beck et al., 2021; McKerracher & Núñez-de la Mora, 2022). Methods like the Toolbox Dialogue Initiative can even provide structured sets of questions to help researchers identify and critique their underlying values and assumptions (Hubbs et al., 2020).

Organized reflection can have powerful effects on how science is interpreted and disseminated. For example, if an advisory committee is formulating a scientific report, organized reflection about the values (including the interests) of the authors can help the organizers to make sure that there is sufficient diversity of perspectives among the authors to help uncover subconscious value influences. Similarly, if journal editors recognize the range of values that authors and peer reviewers are likely to bring to an article, they can take better steps to ensure that the peer review process is conducted by reviewers who bring diverse perspectives and can uncover potential subconscious value influences on the research. Importantly, even when these approaches do not convince specific scientists that they are being subconsciously influenced by particular values and that they need to disclose those values (Tier 1), these approaches still increase the chance that other scientists will recognize that values are likely to be playing a role and put in place strategies to balance them with alternative perspectives (Tier 2).

Based on the considerations raised in the previous sections of this paper, we suggest that in many contexts (e.g., required disclosures for publications or advisory committees), it may be more productive for organized reflection to focus on *interests* rather than on *values in general.* There are two reasons for this. First, contextualized, concretized interests, rather than abstract values, may have more powerful impacts on scientific practice. Second, focusing on interests narrows the scope of discussion so that it is more manageable. In this regard, it is important to counter the view that disclosing non-financial conflicts of interest in the context of publications is undesirable because they are too widespread and too difficult to measure (Bero & Grundy, 2018).^16^ Our view is that people can reasonably be asked to reflect on, disclose, and discuss: 1) whether there are *circumstances* that convert values into interests (both financial and non-financial); and 2) whether such interests have the *potential* to influence scientists’ judgment and behavior in a particular circumstance.

### 6.2. Open Science

A second approach for enhancing the effectiveness of systemic value-management approaches so that they better address subconscious values is to foster openness not only about values (through organized reflection) but also about the scientific research process itself (see Elliott, 2022a). The Open Science Movement has emerged as an extensive effort to foster various forms of openness in science (Center for Open Science, 2024; Leonelli, 2023; Orion, 2024). Open science activities can play a crucial role in addressing the subconscious influences of values because they can facilitate more effective transformative criticism of the sort that Longino (1990, 2002) recommends. Specifically, if scientists have a more detailed understanding of how others have reasoned from their data to their conclusions, they are better equipped to identify questionable inferences that could be grounded in value-laden background assumptions or other subconscious value influences. In some cases, value-laden assumptions may become evident (Tier 1), but even if the values themselves are not identified, the resulting influences on science can be made visible (Tier 2).

As noted above, Elliott (2017) has previously called for openness in order to manage value influences (see also Douglas, 2008; Stanev, 2017). However, others have expressed caution about utilizing this approach to managing values because of various concerns: (1) it might not be sufficiently effective as a management strategy; (2) it could confuse members of the public and generate unjustified skepticism; (3) the release of private or sensitive information could cause harm of various sorts (see e.g., John, 2018; Quinn, 2021; A.S. Schroeder, 2021; Winsberg, 2025). Recognizing the potential for subconscious value influences helps to clarify why it is important to open scientific processes up to scrutiny, even if this may need to be limited in some respects. In order to equip scientists to critique each other’s work effectively while not knowing how values (as well as other psychological factors) might have subconsciously influenced the work, they need access to as much detailed information as possible about each other’s reasoning. This is part of why those who seek to address financial COIs in the chemical industry (including in the context of debates about EDCs) typically argue that it is problematic for industry studies to remain unpublished and unavailable to the general public (Michaels, 2008). Even though these studies may not be entirely trustworthy, they are still used as the basis of public communication, policy advocacy, and regulatory decision-making. Without having access to the details of these studies, it is difficult to determine how conscious or subconscious values might have influenced them.

Ideally, open science practices would provide for the availability of all the information needed to assess the quality, integrity, and validity of research. This could include methods and materials (e.g., chemical reagents and cell lines), computer code, data, documents, and other types of information needed to reproduce or evaluate research. For example, many journals now require researchers to deposit their data on public websites as a condition of publication (Resnik et al., 2019). Other open science practices (e.g., open access publications, preprints, and preregistration of study designs) can assist with transformative criticism of research in additional ways (Elliott & Resnik, 2019). Admittedly, open science practices can also have weaknesses, such as limiting epistemic diversity by forcing researchers to employ standardized approaches for sharing information (Leonelli, 2023; Santana, 2024), but these concerns need to be weighed against the benefits of these practices and managed appropriately. Recognizing the important role that open science can play in addressing subconscious values adds weight to the view that these weaknesses should be managed rather than being used to reject open science practices entirely.

### 6.3. Effective peer review

Peer review plays an important role as a venue for the transformative criticism that Longino (1990, 2002) envisions. Peer review helps to manage both conscious and subconscious value influences because it is a system in which scientists can critically evaluate each other’s work and uncover crucial methodological judgments or shortcomings as well as other influences on research (Shamoo & Resnik, 2022). Importantly, even when it is difficult to identify specific values that caused particular methodological judgments, peer reviewers can still identify the *influences* of those values (e.g., methodological choices) and encourage reflection about them. In these regards, peer review plays a complementary role alongside open science in managing values in science. However, even though peer review has been a key pillar of scientific publishing since the 1800s, it is no guarantor of high-quality science (Lee, 2015). For example, since reviewers usually do not review original data or research protocols, it is often difficult for them to detect problems with the integrity of the data, such as fabrication or falsification. Also, reviewers and editors are themselves susceptible to bias, error, and incompetence (Shamoo & Resnik, 2022).

A number of steps can be taken to strengthen the effectiveness of peer review. For example, policies can be implemented to help manage reviewers’ own COIs and educate reviewers about effective reviewing practices. This includes educating both authors and reviewers about their own subconscious values and alerting reviewers to the fact that researchers who disclose interests are only disclosing those they are aware of. Journals should also continue to experiment with different forms of peer review (Lee & Moher, 2017), such as double-blind and open review (Resnik & Elmore, 2016), with a particular focus on determining which approaches are most conducive to helping authors and reviewers reflect on potential subconscious value influences. Post-publication peer review can also be an important part of this process not only because it continues the dialogue and criticism that is essential for progress (Hunter, 2012) but also because opening up work to a wider range of people makes it more likely that subconscious biases and their effects will be detected. Finally, it is important for scientists, journals, and institutions to take appropriate steps to correct the scientific literature when problems with published papers are detected. Papers that are retracted due to unreliable or fabricated data, or other serious problems, should be clearly marked and linked to retraction notices in databases. Retraction notices should be as clear and detailed as possible so that scientists can understand the reason for retraction which may, at times, include failure to recognize and manage subconscious biases (Vuong, 2020).

### 6.4. Recusal and limitations

Once subconscious values and/or their effects have been made visible through strategies such as organized reflection, open science, and peer review, it may be advisable in some cases to prevent researchers who are subject to particularly significant value influences from engaging in some scientific activities. In the COI literature, it is broadly recognized that disclosure is seldom sufficient to manage the potential adverse impacts of COIs on scientific judgment and decision-making (Elliott, 2008; De Melo-Martín & Intemann, 2009); therefore, it may be necessary to require a person to recuse themselves from the situation that creates the COI or limit their activities that could be negatively impacted by the COI (Shamoo & Resnik, 2022). Consider some examples of this strategy.

1. Most funding agencies prohibit scientists to review grant proposals submitted by collaborators, former students or advisors, or even colleagues at the same institution (Shamoo & Resnik, 2022).
2. Many journals do not allow scientists to review manuscripts if they have a significant COI (such as a financial interest or a case where the authors are from a competing laboratory) (Shamoo & Resnik, 2022).
3. When a member of an institutional review board (IRB) has a significant role (such as principal or associate investigator) on a protocol submitted to the committee, they may be required to recuse themselves from voting on the protocol, but they may be asked to provide the committee with information about it and answer questions.
4. Some scholars are recommending that industry scientists not participate directly (although they could serve as “observers”) on a new intergovernmental science-policy panel currently being developed to study chemicals, waste, and pollution prevention (Schäffer et al., 2023).

The strategy of recusal or limitation can help address the concern (mentioned in Section 4) that individual scientists who are affected by subconscious values can still cause a great deal of social harm, even when a system is in place for their claims to be challenged by other scientists. Preventing these individuals from participating in influential activities can help lessen—although not completely eliminate—the potential for harm. This approach also accords with the guidance of some psychological researchers who argue that the only fully effective way to manage COIs is to prevent those with significant conflicts from making decisions affected by the conflicts (Chugh et al., 2005). This line of reasoning can be applied to values more generally. Once scientists’ subconscious values have been rendered visible (either by the scientists themselves or by others who bring the values to light), a wide range of steps can be taken to limit the activities of those who might be affected by them. For example, in 2013, a number of journal editors published a set of highly opinionated editorials in response to proposed European Commission (EC) regulatory policies regarding EDCs (Dietrich et al., 2013). These editorials were challenged by other scientists (Gore et al., 2013), and investigative journalists found that many of the journal editors had undisclosed COIs (Horel & Bienkowski, 2013). In cases where a journal editor has such a strong opinion about a debated scientific issue, in addition to COIs, it would be advisable for them to recuse themselves from making decisions about papers on that topic.

### 6.5. Ethical, legal, and regulatory oversight

In order to make all the aforementioned strategies operate effectively, they need to be embedded in an organized system of norms, policies, procedures, guidelines, and/or rules that take seriously the significance of subconscious values (see Resnik & Elliott, 2019, 2023). These can be instituted by a variety of different organizations, including universities, journals, funding agencies, companies, nongovernmental organizations, and government legislative bodies. Asking people to disclose and reflect on subconscious values or interests, implement open science practices, strengthen peer review, and/or recuse themselves from activities all require ethical, legal, and regulatory oversight. While most scientists are honorable, ethical, and professional, not all are aware of (or enthusiastic about revealing) their cognitive vulnerabilities, and best practices for open science, peer review, and reorganization of scientific roles and processes (e.g. recusals) can be time consuming and demanding. Serious ethical problems with science, such as data fabrication or falsification, plagiarism, violations of human or animal regulations, financial fraud, inappropriate authorship, violations of confidentiality of peer review, mismanaged COIs, and harassment or bullying are, unfortunately, realities of modern science (Shamoo & Resnik, 2022) and demonstrate that rigorous oversight, incentives, and disincentives might be needed to ensure that policies, procedures, guidelines, and rules are followed.

## 7. Conclusion

By examining the relationship between values and interests and reviewing evidence concerning the impact of COIs in science, we have highlighted the potential for values to exert subconscious influences on scientific judgment. We have argued that most contemporary proposals for managing values in science are not well-designed for handling these subconscious value influences. In response to this weakness, we suggested an initial set of management strategies that could prove fruitful for addressing the subconscious ways in which values influence science. We endorsed the systematic approach championed by Longino (1990) and others (e.g., Solomon, 2001), but we argued that this framework should be augmented with policies for promoting effective reflection, openness, peer review, and elimination of particularly severe value conflicts, along with ethical, legal, and regulatory oversight. Future work on science and values should explore in greater detail these strategies that researchers, their employers, funders, journals, and regulatory bodies can use to manage subconscious values and their influences.

## Figures and Tables

**Table 1:** Values and interests in scientific research.

Value	Circumstance that gives rise to interests	Interests (things we prefer/desire in a particular context)
Economic prosperity	Ownership of stock in a company that sponsors one’s research	Financial success of the company in which one owns stock
	Receiving consulting or speaking fees from a company that makes products related to one’s research	Ongoing relationships and financial opportunities with the company
	Ownership of patents related to one’s research	Financial benefits associated with the patents
Career advancement	Serving on a committee that is reviewing one’s research (such as a human research ethics committee)	Seeing one’s research approved by the committee
	Reviewing a paper submitted by a competing laboratory (with both laboratories working on the same research problem)	Slowing down or obstructing publication by the competitor
Professional and personal relationships	Reviewing a grant application submitted by a colleague at one’s institution or a collaborator, former student, or former advisor	Seeing one’s grant application approved
	Conducting an economic analysis of a health technology that could benefit a family member who has a particular health condition	Seeing a medicine funded that could benefit the family member
Moral, political, or social concerns	Consulting (without pay) or serving on the board for a nongovernmental organization (such as an environmental or public health group) that advocates for policies related to one’s research	Supporting the NGO’s interests

## Data Availability

No data was used for the research described in the article.

## Footnotes

1 By ‘integrity’, we mean adherence to science’s epistemic and ethical norms, such as honesty, rigor, reproducibility, objectivity, openness, protection of human and animal subjects, and social responsibility (Shamoo & Resnik, 2022). There are numerous examples in the history of science in which value-related influences have corrupted or impeded science, such as the Catholic Church’s punishment of Galileo, Lysenkoism in Soviet science, research on race and intelligence, eugenics, and suppression of data on adverse effects of drugs by pharmaceutical companies (see Resnik, 2007, 2009).

2 The words ‘subconscious’ and ‘unconscious’ have different meanings in scientific and lay vernacular. In this paper, ‘subconscious influence’ refers to psychological influences on judgment, decision-making, reasoning, or perception that one is not directly aware of, but which one could become aware of upon further reflection. An ‘unconscious influence’ refers to an influence that is so deeply ingrained in one’s psychology that they cannot become aware of it without considerable effort (Seltzer, 2019). For example, racial, ethnic, and gender biases may be unconscious (Devlin, 2018). It is possible that some influences of values on science are unconscious, but most of the management strategies that we suggest here are applicable to either subconscious or unconscious value influences.

3 Elliott and Korf (2024) acknowledge that these four concepts, although distinct, overlap in significant ways. So, for example, a belief about what is desirable could also serve as a criterion for choice or as a causal factor that influences a scientist’s reasoning.

4 There have also been debates historically about whether interests can all be reduced to economic concerns, whether they are fixed or changeable and contextual, and whether they are held by individuals or collectives.

5 According to many political and moral theorists, the function of moral and legal rights is to protect interests, such as interests in life, liberty, or property (Gewirth, 2001). Also, the concept of harming is usually understood as doing something that negatively impacts a person’s interests (Feinberg, 1973).

6 These circumstances are sometimes referred to as ‘dual agency dilemmas’ or ‘conflicts of commitment’, but they have the same structure, consequences, and management strategies as conflicts between self-oriented interests and other-oriented interests.

7 Although ‘values’ and ‘interests’ can refer either to the things that are desired or to the mental state of desiring them, we will focus on the mental state of desiring them because those mental states, rather than the things themselves, are what can influence scientists’ judgment. Moreover, although one could discuss whether particular values or interests are normatively *desirable* as opposed to being merely descriptively *desired,* in the scientific contexts that we are considering it is generally sufficient to focus on their usage in a descriptive sense, as things that are desired by individuals or groups.

8 EFSA recently changed course and proposed regulating BPA at much lower levels.

10 Our focus in the remainder of this paper will be on managing *non-epistemic* values because they have been central to the recent literature on values and science, but we think that even *epistemic* values (e.g., simplicity, explanatory power, scope) could have subconscious influences that merit additional discussion. Epistemic values have been treated as relatively unproblematic in the recent literature, but it is plausible that a scientist who valued explanatory power particularly highly could, for example, display motivated cognition like confirmation bias and assess the evidence for an explanatorily powerful theory more favorably than the evidence warranted. Thus, the argument that we are making in this paper raises new concerns about the roles that epistemic values play in science.

11 Douglas does not distinguish between epistemic and non-epistemic values in her 2009 book, although she does distinguish between values and “epistemic criteria,” such as internal consistency. See Douglas (2013) as well for her perspective on “cognitive” values.

12 We used a double-negative here because in frequentist statistics, scientists do not accept hypotheses per se but reject null hypotheses. So, to “accept” the hypothesis that a drug is safe at a particular level, one would reject the null hypothesis that it is unsafe. A p-value is the probability that a result is due to chance (Forthofer & Lee, 1995).

13 In more recent work, Douglas (2021) has adopted more complex approaches to managing values that incorporate other elements in addition to the direct/indirect role distinction.

14 Elliott (2022b) argues that transparency can take many forms, and so he contends that scientists can provide useful information about their data, methods, and analytical approaches (which can allow others to identify value influences on their work) even if they are not transparent about their values. We will return to this issue of transparency and open science when we provide our recommendations.

15 Although it goes beyond the scope of this paper, there is much work to be done in order to clarify how to implement and apply Longino’s four desiderata for the scientific community, which can stand in tension with one another (Borgerson, 2011; see also De Melo-Martrn & Intemann, 2018).

16 In response to the concern that non-financial interests are too ubiquitous to disclose, we would argue that this reflects a failure to distinguish clearly between values and their more specific instantiations as interests.
